# Supplementary material for: Donor-Specific Cell-Free DNA qPCR Quantification as a Noninvasive Accurate Biomarker for Early Rejection Detection in Liver Transplantation
Source: J Clin Med. 2022 Dec 21;12(1):36. doi: 10.3390/jcm12010036 (PMC9821236; doi:10.3390/jcm12010036)
Supplement: Supplementary file 1 [file jcm-12-00036-s001.zip › jcm-2084626-supplementary.pdf]

Supplementary Table S1. Causes of patient death

Nº patient	Time to rejection (days)	Time to death (months)	Causes of death
**65**	420	14	Graft dysfunction and multiple organ failure after ductopenic rejection and graft dysfunction
**125**	10	34	Respiratory failure due to pneumonia
**181**	-	4days	Subarachnoid hemorrhage, familial amyloidotic polyneuropathy.
**227**	-	14	Multiple organ failure as a consequence of shock septic (*Candida albicans*)
**253**	-	3	Multiple organ failure as a consequence of shock septic (*cytomegalovirus* infection)
**278**	-	7	Multiple organ failure as a consequence of shock septic (intra-abdominal origin, *Pseudomonas aeruginosas*)
**286**	-	3	Long period at the ICU with severe complications. Multiple organ failure as a consequence of shock septic (abdominal origin, *Escherichia coli* and *Candida albicans*)
**308**	-	27	Respiratory failure due to Microcytic Lung Carcinoma progression
**319**	-	31	Liver failure due to autoimmune hepatitis

Supplementary Table S2. Comparison between stable patients and individual suffering any hepatic damage

	Stable (n=75)	Hepatic damage (n =22)	p
**Age (years)**	56 [22 - 71]	51 [18 - 68]	**0.05**
**Sex (male)**	35 (46.6%)	15 (68%)	0.6
**Warm Ischemia Time (min)**	30 [12.5-60]	32.5 [22-60]	0.84
**Cold Ischemia Time (min)**	382.5 [270-551]	360 [250-430]	0.09
**Previous ICU**	5 (6.6%)	5 (23%)	**0.04**
**Previous ICU time (days)**	3 [2 - 58]	1 [1 - 34]	0.29
**ICU post-TX (days)**	6 [3 - 28]	6 [4 - 54]	0.09
**Hospitalization (days)**	16 [4 - 100]	29.5 [11-109]	**0.0002**
**Beta-globin 48h (GKE/mL)**	319.6 [104-2626]	374.3 [104-4979]	0.52

Continuous values expressed as median [range]. Beta-globin values are the mean of 48h values after TX. GKE: Genomic Kilo Equivalent. Mann-Whitney U-test was used to compare differences for all continues parameters studied with exception of cold ischemia that was normally distributed (T-test); Bold: significant p values ($p < 0.05$)

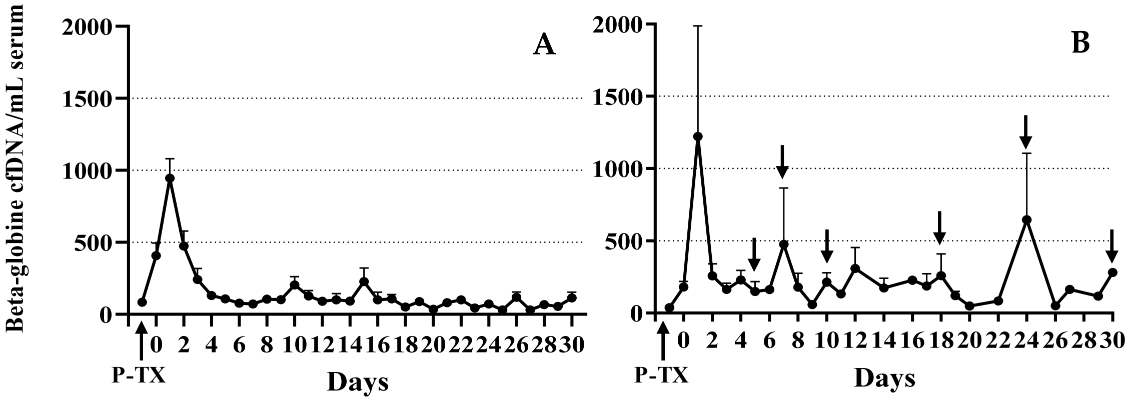

Supplementary Figure S1. Total cfDNA levels during the first month post TX. Decrease of total cfDNA levels from day 0 of TX, measured by beta-globin amplification (KEG/mL). A) Patients who accepted transplanted livers with no rejection; B) Patients with BPR during the first month after TX. Data expressed as mean + SEM. P-TX: pre-transplantation sample before organ reperfusion.

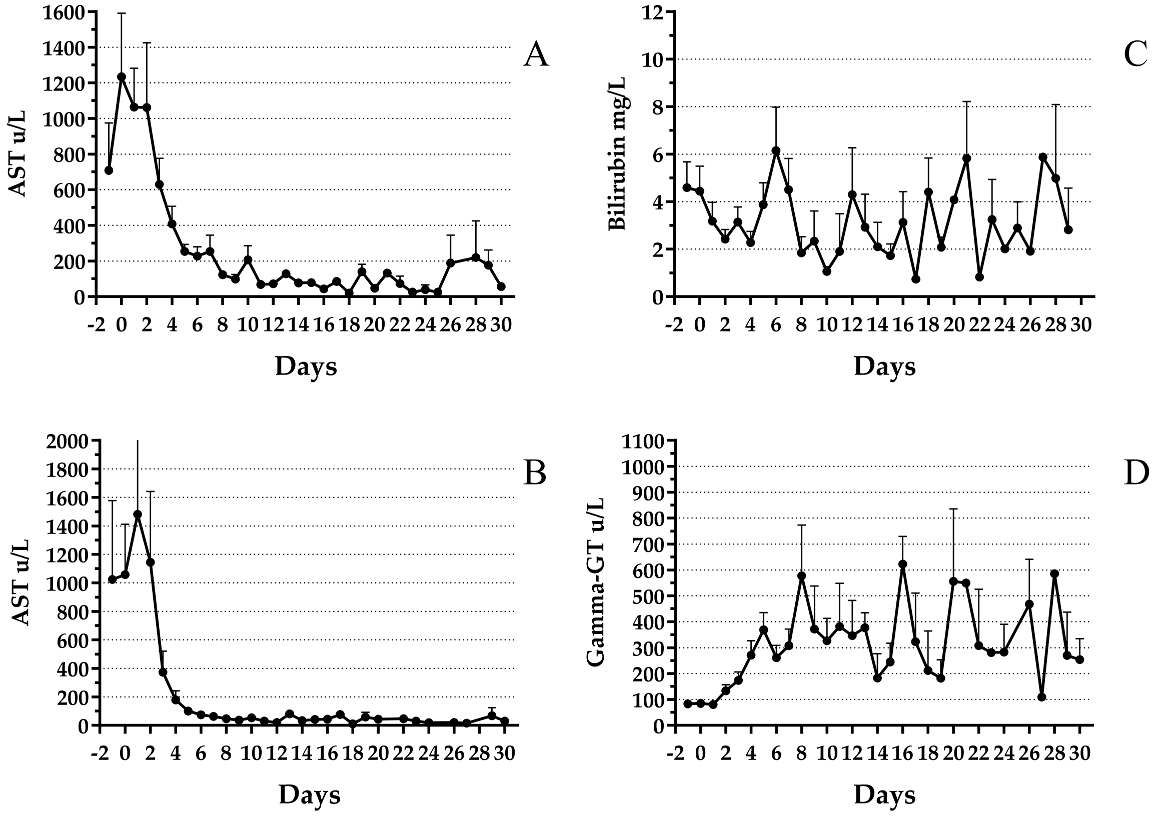

Supplementary Figure S2. Function liver markers levels during de first month post TX. Decreasing levels of liver function markers from day 0 of TX. A) Glutamate-Pyruvate Transaminase (GPT) levels; B) Glutamate-oxaloacetate transaminase (GOT) levels; C) bilirubin levels; and Gamma-glutamyltransferase (Gamma-GT) levels. Data expressed as mean + SEM.
